# Validity of computed tomographic measurements and morphological comparison of cubital tunnel in idiopathic cubital tunnel syndrome

**DOI:** 10.1186/s12891-020-3108-9

**Published:** 2020-02-05

**Authors:** Sang Ki Lee, Seok Young Hwang, Won Sik Choy

**Affiliations:** Department of Orthopedic Surgery, Eulji University College of Medicine, 1306 Dunsan-dong, Seo-gu, Daejeon, 302-799 South Korea

**Keywords:** Ulnar nerve compression neuropathy, Idiopathic cubital tunnel syndrome, Bony structure variation, Computed tomography, 3D modeling

## Abstract

**Background:**

Ulnar neuropathy is a common reason for referral to hand surgeons, and 10 to 30% of cubital tunnel syndrome (CuTS) is idiopathic. We hypothesized that the cause of idiopathic CuTS is in the bony structure.

**Methods:**

We analyzed 79 elbows (39 idiopathic CuTS and 40 without CuTS symptom) using computed tomography and Materialize Mimics software to compare the differences between the two groups. We proposed a new bony cubital tunnel with a new boundary that could play a role in ulnar nerve compression symptom.

**Results:**

The mean cubital tunnel volume was 1245.6 mm^3^ in all patients, 1180.6 mm^3^ in CuTS patients, and 1282.3 mm^3^ in the control group. A significant difference (*p* = 0.015) between two groups was found. Bony cubital tunnel cross-sectional area, cubital tunnel depth, and cubital tunnel angle also showed significant differences.

**Conclusion:**

The shape of the bony cubital tunnel is an important cause of CuTS, and the normal variation of the volume and cross-sectional area of the cubital tunnel and cubital tunnel angle could influence the occurrence of idiopathic CuTS.

## Background

Cubital tunnel syndrome (CuTS) is one of the most frequently occurring compression neuropathies in the upper extremity [1, 2]. Causes of CuTS include elbow osteoarthritis, constriction of the cubital tunnel retinaculum, medial elbow ganglions, ulnar nerve subluxation, contusion, and cubitus varus or valgus deformities. However, 10 to 30% of cases are idiopathic [3].

The ulnar nerve originates from the ulnar sulcus and enters the cubital tunnel posterior to the medial epicondyle and medial to the olecranon and runs between the ulnar and humeral heads of flexor carpi ulnaris [4]. Previous studies demonstrated that soft tissues, such as retinaculum, fibrous band, and anconeus, cause ulnar nerve compression [1, 3, 5, 6], whereas other studies showed that the bony structure [7–9] causes strain of the ulnar nerve that runs directly behind the medial epicondyle constituting the boundary of the cubital tunnel during elbow flexion [10]. However, no studies on the association of the shape of the bony structure with CuTS symptoms have been conducted. Thus, we speculate that idiopathic CuTS could be attributed to the compression or strain of the ulnar nerve that is triggered by the shape of the bony structure in the absence of arthritic change, instability, and deformity or space-occupying lesion.

**Fig. 1:**
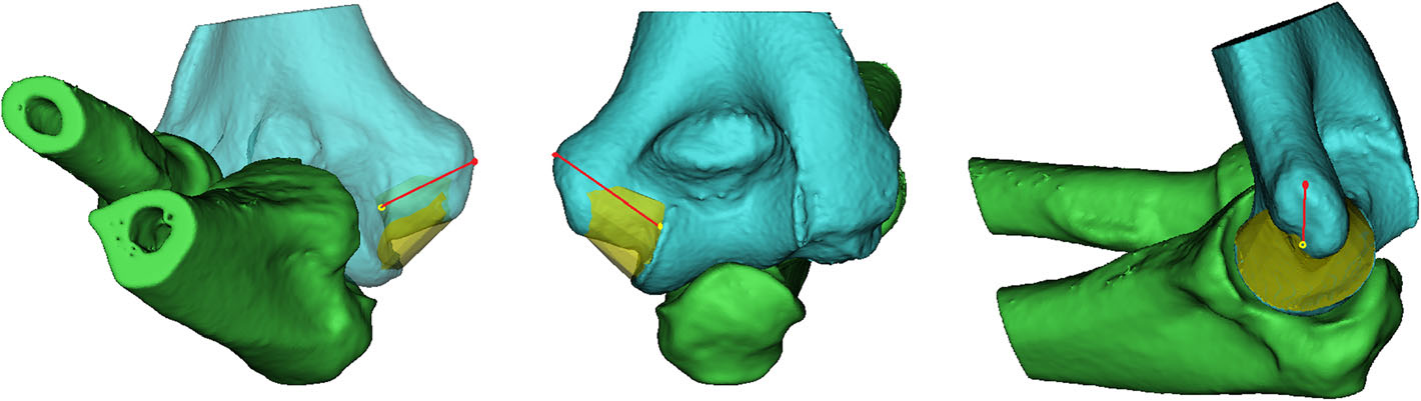
The elbow, reconstructed by Materialize Mimics software using CT images. Yellow area represents the cubital tunnel; red dot, apex of the medial epicondyle (AME); yellow dot, the center of trochlear border (CTB). The red line connecting the two points represents the vertical axis of bony cubital tunnel (ABT)

Previous studies have measured cubital tunnels by transforming them into curves centered on the medial epicondyle. It was reported that the tunnel is short and the space decreases when the elbow is flexed at 135° [5]. In the present study, the bony cubital tunnel was assumed to be a semi-circular tunnel with a line connecting the center of the trochlear and the medial epicondyle (Fig. 1), and the bony structure was analyzed in the elbow full flexion state.

Measurement of the bony structure using computed tomography (CT) accurately reflects true object dimensions with minimal errors [11] and three-dimensional (3D) volume rendering is useful to predict postoperative outcomes [12] and postoperative bone structure [13, 14]. The bony structure is shown more accurately with CT than with magnetic resonance imaging (MRI) because of the higher contrast between the cortical border and surrounding soft tissues in CT [15, 16]. Moreover, in CT, high-resolution images are easily attainable and capturing of images of a larger area is possible. In MRI, the resolution depends on the magnetic field intensity, and distortion is generated when the field of view is large [17]. Besides, the CT has advantages of 3-dimensional analysis of structure comparing with that of MRI in terms of fabrication of better patient specific instrumentation [18].

Hence, the purpose of this study was to investigate the relationship between radiographic parameters based on CT and symptom of idiopathic CuTS. We hypothesized that CT-measured parameters of bony cubital tunnel are related to idiopathic CuTS symptom.

## Methods

This study was approved by our Institutional Review Board. Data from all patients treated during the study period are available for review and analysis. All participants provided informed consent prior to data collection. This study used a convenience sample of individuals attending clinics at our university hospital and consecutive patients that met the inclusion criteria were invited to participate in this study.

### Patients

Among the patients with CuTS who visited our institution for treatment between April 2010 and August 2017, we excluded those with secondary associated pathologies. Thirty-nine patients with idiopathic CuTS (18 male, 21 female; mean age, 47.8 years (SD 11.8)) were assigned to group A, and 40 patients without CuTS symptom (20 male, 20 female; mean age, 42.5 years (SD 8.8)) were assigned to group B (control).

Group A included patients with ulnar nerve injury (McGowan grade 2, *n* = 31; McGowan grade 3, *n* = 8) with muscle weakness, pain, numbness, or paresthesia in the distribution of the ulnar nerve and a positive result in Tinel’s nerve percussion test and electromyography. An experienced neurologist performed all examinations and revealed a substantially delayed motor nerve conduction velocity in the ulnar nerve segment crossing the elbow. Twenty-eight McGowan grade 2 patients and 8 McGowan grade 3 patients underwent cubital tunnel release, and all patients had symptom improvement. Three McGowan grade 2 patients who were lost to follow-up were excluded.

Group B (control) included patients without symptoms of CuTS who visited the hospital because of elbow pain that required CT imaging (in group A, CT was performed for simple elbow joint pain); both groups were matched for age and sex. None of the patients had systemic diseases that might have contributed to the occurrence of neuropathy or more proximal compression lesions.

### Imaging techniques

#### Patient positioning

**Fig. 2:**
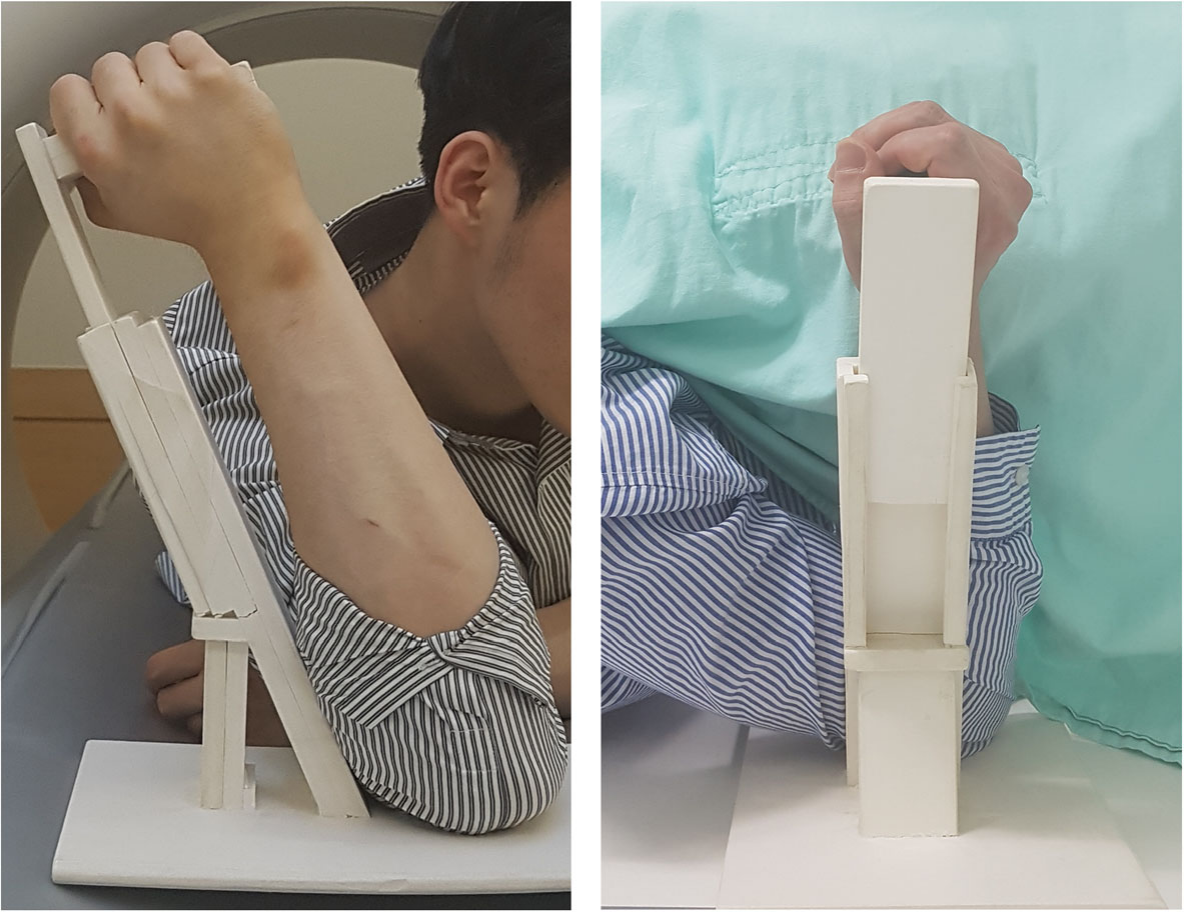
Patient with arm support system in CT machine. The center of the plate of the arm support system is located at the center of the patient’s bed. This arm support system can lengthened to ensure equal elbow flexion angle

Measurement with CT may vary because of tilt vibration resulting from changes in the posture of patients or varied positions of the elbow inside the machine [19]. Thus, an arm support system was prepared for the capturing of images in all patients, ensuring consistent positioning inside the CT machine; adjustments were made for consistency with the posture during x-ray imaging of the cubital tunnel. The arm support system consisted of a tilted wooden bar (20°) and a handgrip on a flat wooden plate. Subjects were positioned nearly prone on the device and were asked to grab the arm support system with the forearm supinated, shoulder externally rotated at 20°, and elbow full flexed (Fig. 2).

### CT imaging data acquisition

CT scan was performed with 128-multidetector computed tomography scanner (SOMATOM Definition AS+, Siemens Healthcare, Forchheim, Germany). The following image acquisition parameters were used: peak voltage of 140 kVp, tube current adjusted by CARE Dose4D software (Siemens Medical Solutions, Erlangen, Germany), 128 × 0.67-mm detector collimation, 0.7 beam pitch, 0.5-s gantry rotation time, and reconstruction slice thickness of 0.6 mm using a bone kernel. Axial data were reconstructed with 0.6-mm-thick sections at 0.6-mm intervals for sagittal and coronal reformation.

### Radiographic parameter measurements

#### Definition of bony cubital tunnel

**Fig. 3:**
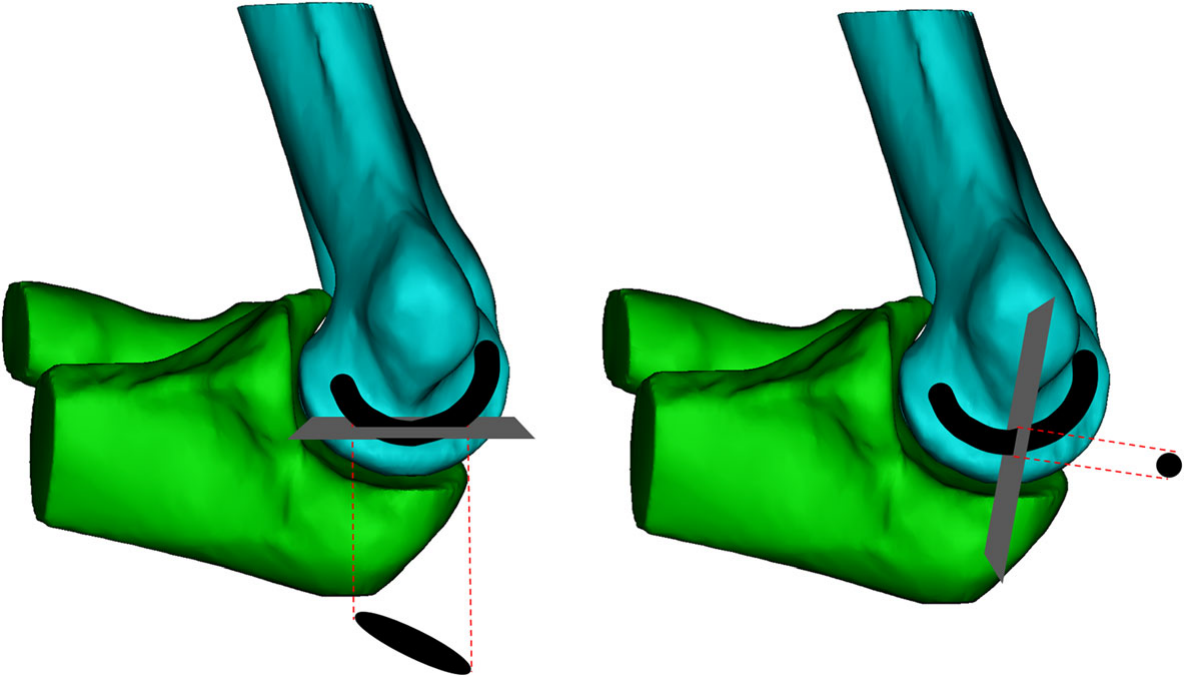
Since the cubital tunnel is a structure forming a curvature, traditional craniocaudally directed axial CT images could not measure the cross-sectional area of the actual nerve passing (left). Cutting the plane vertical to the curvature of the cubital tunnel enables accurate measurement by positive axial image of the curvature where the nerve passes through (right)

Assuming that the cubital tunnel is a semi-circular structure with the line connecting the trochlear and the medial epicondyle as its axis, we could measure the actual nerve passing space. Typical craniocaudally directed axial CT images could not accurately characterize the cubital tunnel (Fig. 3). The bony cubital tunnel in this study was further defined in the following sections.

#### Anatomical landmark

**Fig. 4:**
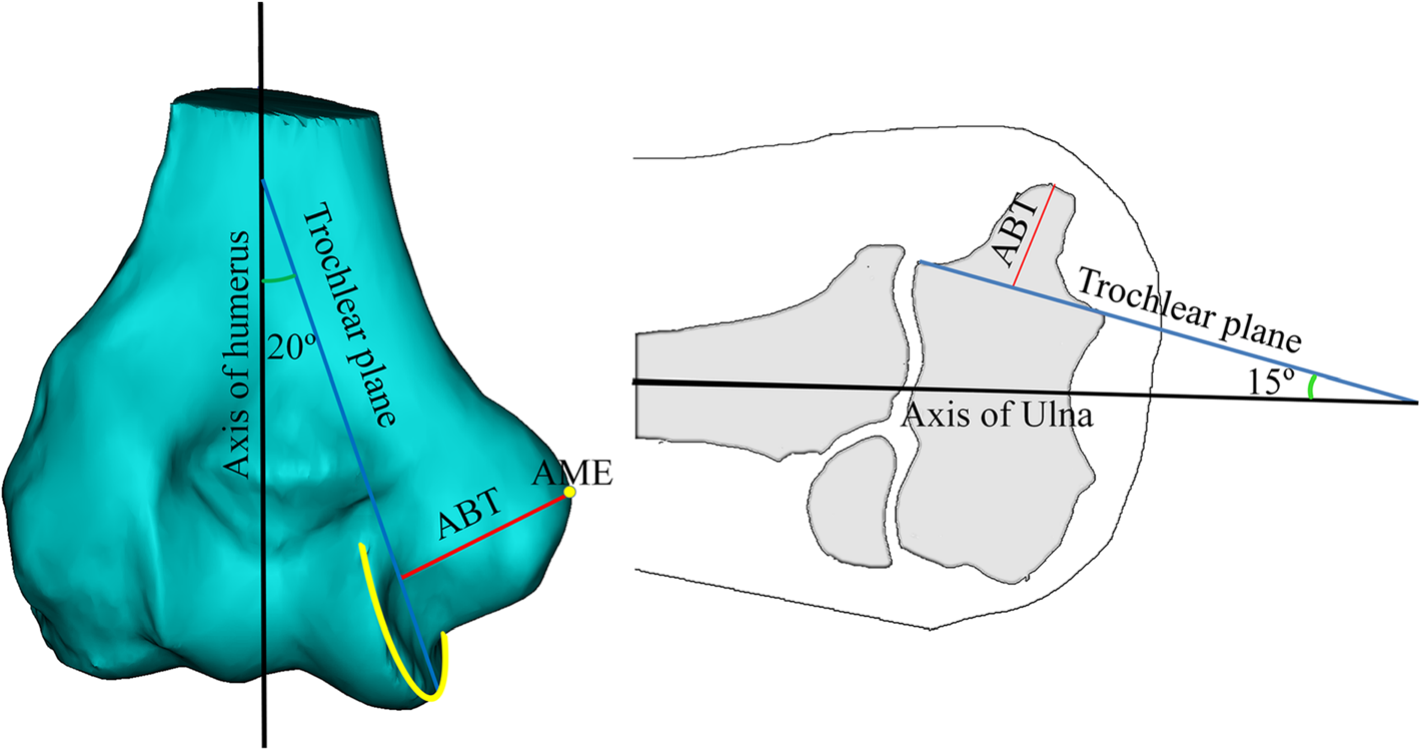
Axis of the humerus is expressed in black line, and the yellow circle in the trochlear plane shows approximately 20° varus (left), with a posterior slope of approximately 15° to the axis of the ulna (black line)

The medial border of trochlear can be expressed as a fan-shaped plane of approximately 20 degrees varus to the axis of humerus and posterior slope of approximately 15 degrees for the axis of ulna (Fig. 4).

**Fig. 5:**
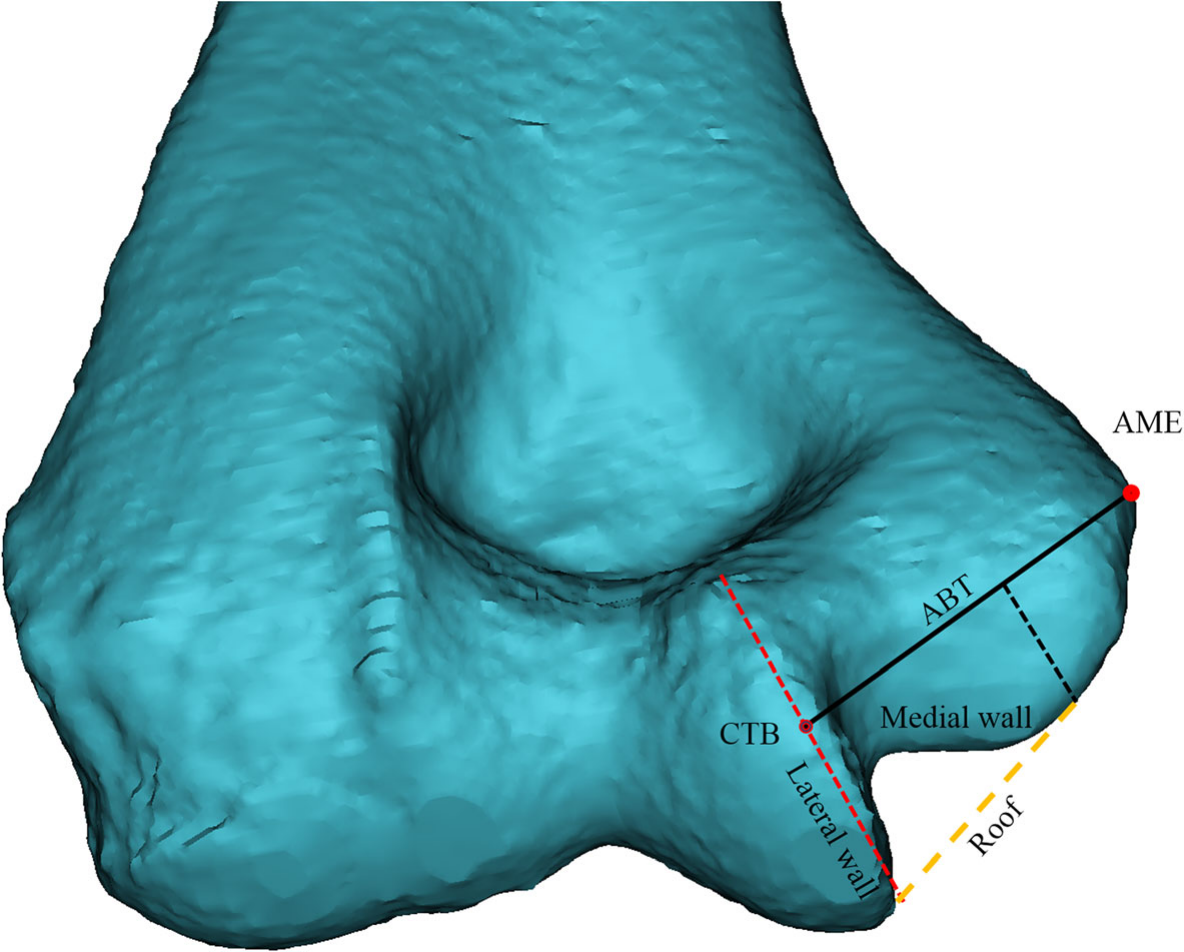
The posterior view of distal humerus. The black line indicate ABT and red dot indicate AME. Medial wall, lateral wall and roof of bony cubital tunnel expressed in the figure

The center of the trochlear articular surface was defined as the center of the trochlear border (CTB), and the point of the medial epicondyle, which was the point farthest to the fan-shape, was designated as the apex of the medial epicondyle (AME). The line connecting AME and CTB was defined as the axis of bony cubital tunnel (ABT), which enabled the measurement of the characteristics of trochlear and medial epicondyle in the bony cubital tunnel. The articular surface of the medial trochlear border became the lateral wall while the medial epicondyle surface constitutes the medial wall (Fig. 5).

#### The roof, entrance, and exit of the bony cubital tunnel

**Fig. 6:**
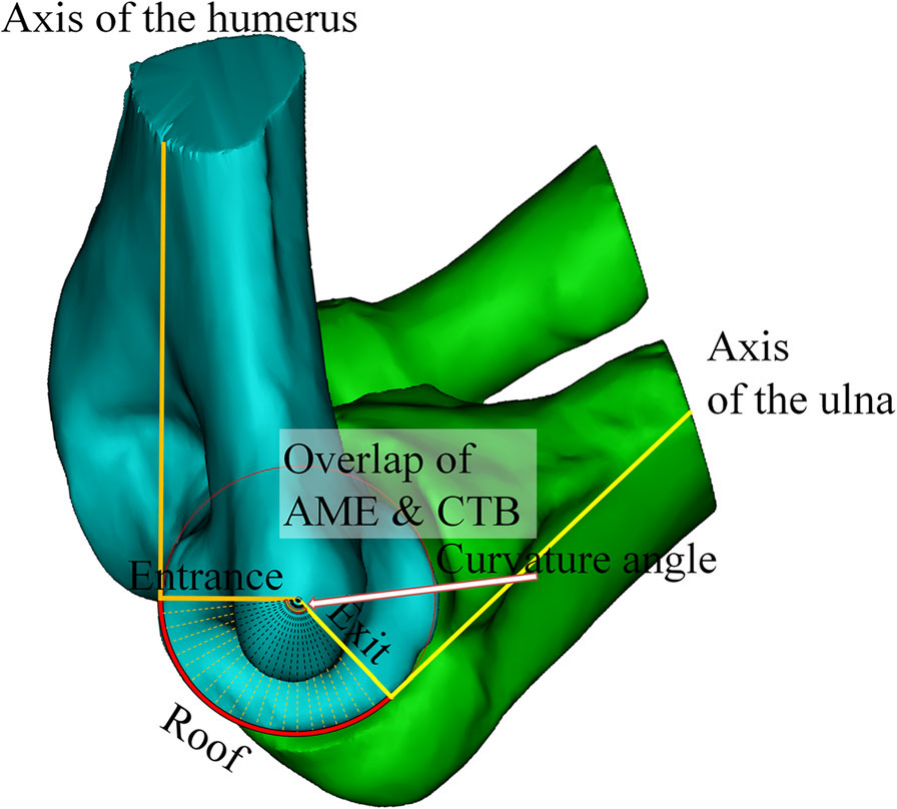
Axis of the humerus is expressed in orange line and ulna is expressed in yellow line. Yellow dotted area represents the roof of bony cubital tunnel. White arrow indicate curvature angle which made of entrance and exit of bony cubital tunnel

The ceiling of the actual cubital tunnel is comprised of Osborne’s fascia; in this study, the plane that was formed from the line connecting the points on the lateral wall and medial wall, which constitute identical degree to ABT, was defined as the ceiling of the cubital tunnel. By taking the course of the ulnar nerve into account, the effect of the bony structure would almost disappear as the trochlear border passes the plane where the axes of the humerus and ulna are located. Therefore, the axis of the humerus and axis of the ulna in the trochlear border were defined as the entrance and exit, respectively. The angle formed by the entrance and exit was defined as the curvature angle (Fig. 6).

#### Bony cubital tunnel volume

Cubital tunnel volume (CTV) was measured by importing raw CT data into the Materialize Mimics 21.0 software (Materialize Interactive Medical Image Control System, Materialize, Leuven, Belgium), and 3D modeling according to the definition of bony cubital tunnel was performed.

#### Cross-sectional area and minimal cross-sectional area angle

**Fig. 7:**
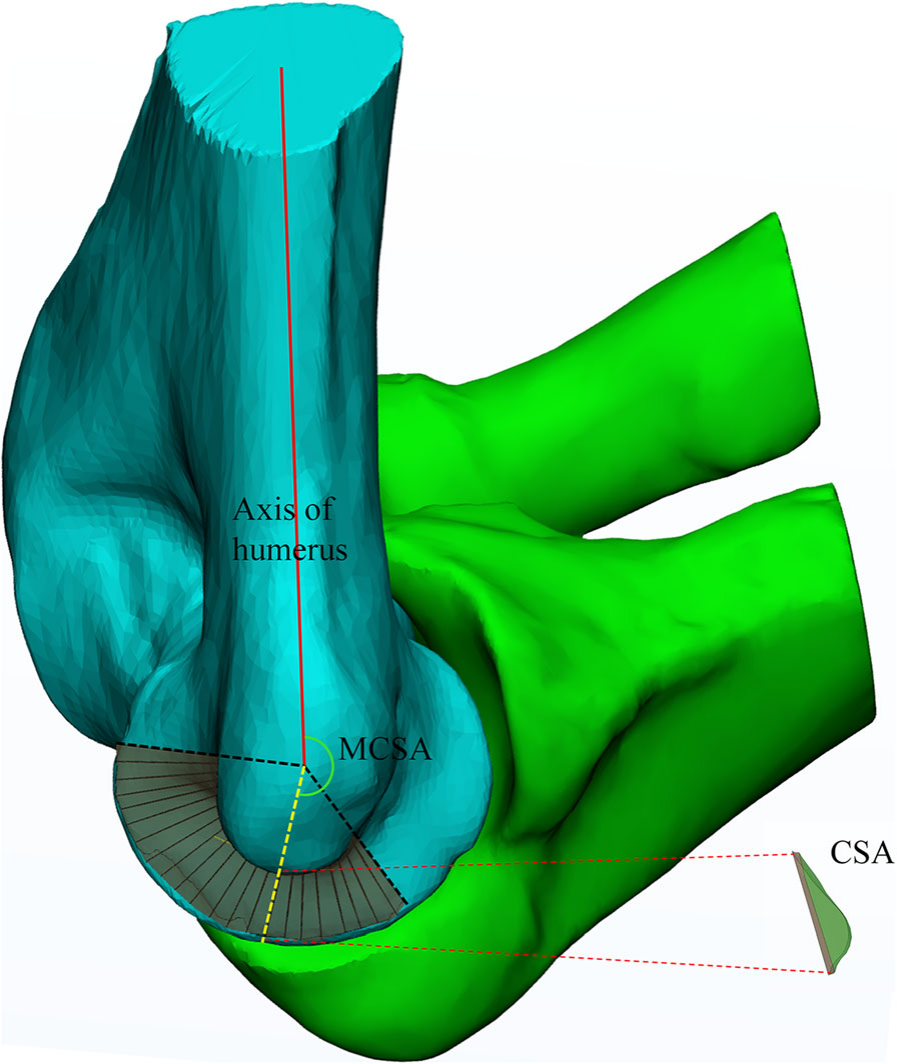
Cross-sectional area of the bony cubital tunnel (through the ABT plane) was measured at every 1° in a clockwise direction from the axis of the humerus, and the smallest cross-sectional angle measured was defined as the minimal CSA angle (MCSA)

Cross-sectional area (CSA) of ​​the bony cubital tunnel (through the ABT plane) was measured at every 1° in a clockwise direction from the axis of the humerus, and the smallest cross-sectional angle measured was defined as the minimal cross sectional area angle (MCSA) (Fig. 7).

#### Cubital tunnel depth and cubital tunnel angle

In this study, we used the image obtained by reslicing at 1° intervals around the ABT in the measurement of cubital tunnel depth (CTD) and cubital tunnel angle (CTA), and we defined the resliced image as a rotatory image (CT syngo Post-Processing Suite software, version VE 36A).

**Fig. 8:**
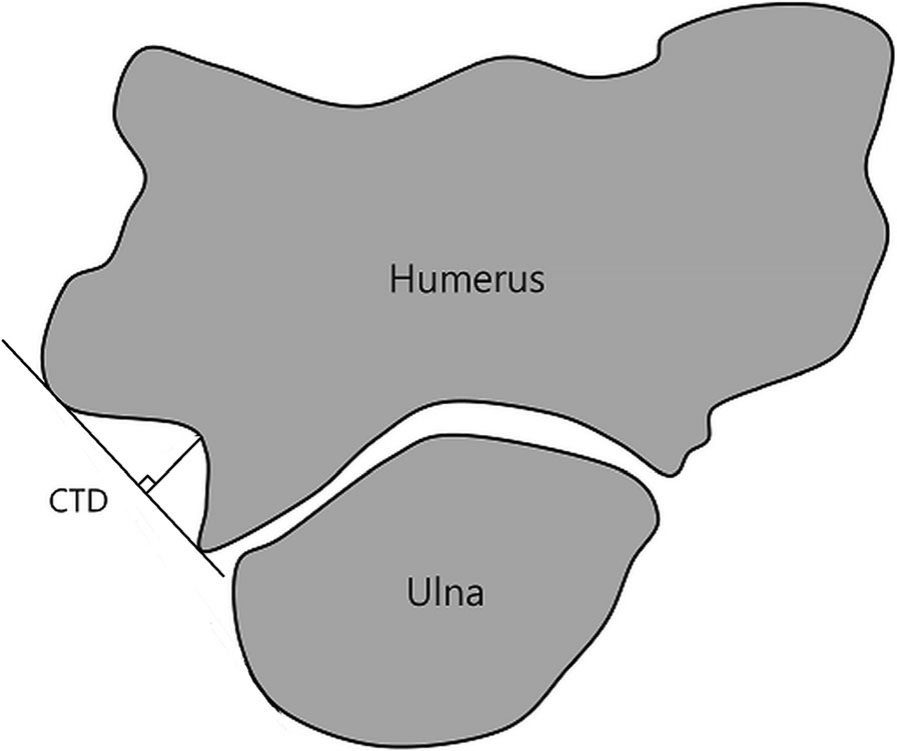
Figure representing CTD

CTD was defined as the length of the line connecting the deepest point of the groove of the ulnar nerve, which is vertical to the line connecting the most prominent part of the inward trochlea and the most prominent part of medial epicondyle (Fig. 8). CTD was measured from each rotary image; the minimum, maximum, and difference between minimum and maximum of CTD were compared as the range of CTA.

**Fig. 9:**
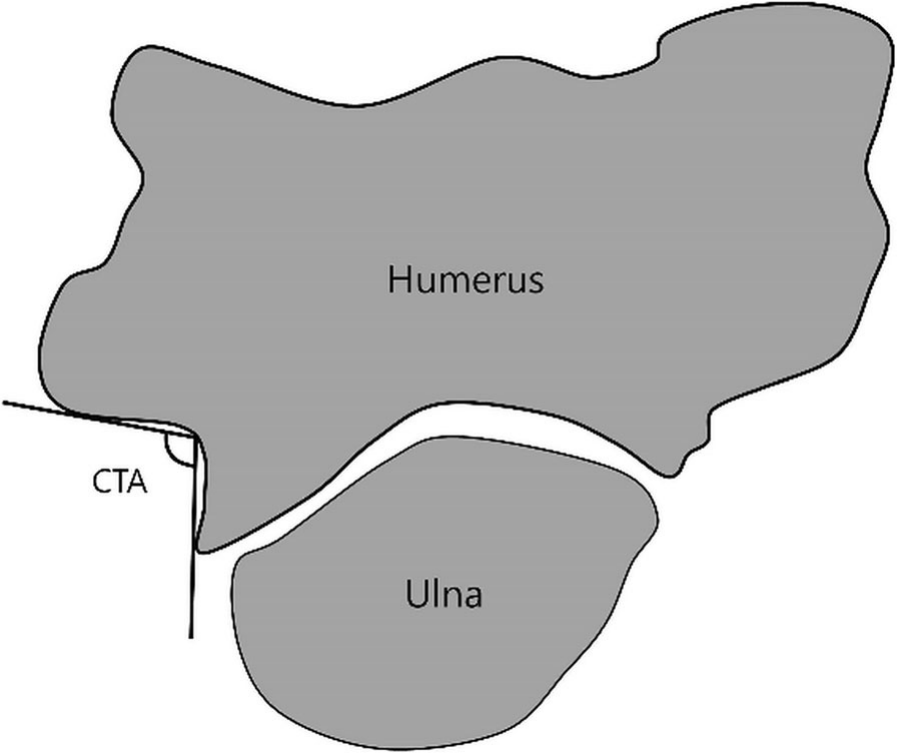
Figure representing CTA

Moreover, CTA refers to the angle resulting from two lines drawn over the medial surface of the trochlea and the inferior border of the medial epicondyle at the deepest point of the cubital tunnel (Fig. 9). CTA was measured from each rotary image; the minimum and maximum CTA and the difference between them were identified. The maximum CTD and CTA values served as the ideal parameter value in cubital tunnel view and the minimum CTD and CTA values represented the narrowest part of the cubital tunnel. In addition, the difference between the minimum and maximum CTD and CTA values indirectly indicates the degree of change in CTD.

We quantitatively measured radiographic parameters by employing the picture archiving and communication system as our image analysis software (Maroview, version 5.4, Marotech, Seoul, Korea). Radiographic parameters was measured by manually delineating with a cursor, and images were evaluated in bone window (width, 2000 HU; level, 500 HU).

### Intraobserver reliability

The measurements were evaluated by two experienced surgeons who were blinded to patient information. To reduce errors, measurements were obtained twice by each of the surgeons, and the average values were calculated. Intraobserver reliability was recorded using the criteria of Winer (degree of bias and mean squared error) [20]. Reliability was classified according to intraclass correlation coefficient as follows: absent to poor (0–0.24), low (0.25–0.49), fair to moderate (0.50–0.69), good (0.70–0.89), or excellent (0.90–1.0). We achieved an intraobserver reliability of 0.94. There were no missing data.

### Statistical analysis

The measured CTV, CSA, MCSA, CTD, and CTA were presented as mean (range). Each radiographic parameter was analyzed using t test. Descriptive statistical analyses were performed using SPSS version 20.0 software (IBM Corporation, Armonk, NY), with an alpha level of 0.05.

## Results

Seventy-nine elbows of 39 patients and 40 healthy individuals were included in this study. Mean age of group A was 47.8 years and that of group B was 42.5 years. There was no significant difference in age, sex, height, weight, BMI, curvature angle between the two groups (Table 1). Also, there was no significant difference between the two groups according to the degree of elbow flexion, affected side, humerus axis and ABT.

**Table 1:** Summary of participant characteristics (mean (SD)) of CTS patient and non-CTS patient groups

	Group A(CTS patient)	Group B(non-CTS patient)	*P* Value
No. of patients, N	39	40	
Affected side (Rt/Lt).	22/17	22/18	.255
Age (yr)	37.0 (8.7)	38.4 (6.6)	.422
Gender, male/female, N	18/21	20/20	.592
Height (cm)	164.4 (9.7)	166.2 (8.2)	.312
Weight (mm)	62.0 (9.7)	69.5 (7.8)	.221
BMI (Kg/m2)	24.7(5.8)	25.4 (4.2)	.826
Curvature angle (°)	135.5 (5.6)	138.7 (4.6)	.281

The mean CTV was 1245.6 mm^3^ in all patients, 1180.6 mm^3^ in group A, and 1282.3 mm^3^ in group B; significant difference between the groups was noted (*p* = 0.015). The mean CSA of the cubital tunnel was 49.6 mm^2^ in all patients, 43.7 mm^2^ in group A, and 52.2 mm^2^ in group B; the difference was statistically significant (*p* < 0.001). Almost all CSA measurements were greater in group B than in group A (Table 2.)

**Table 2:** Radiologic measurement of bony cubital tunnel ^*^

	Bony cubital tunnel volume, mm3	Cross sectional area (CSA), mm2	Minimum Cross sectional area	Minimal cross sectional angle (MCSA), degree	Cubital tunnel depth (CTD) Maxima, mm	Cubital tunnel depth (CTD) Minima, mm	The range of CTD, mm	Cubital tunnel angle Maxima (CTA), °	Cubital tunnel angle Minima (CTA), °	The range of CTA, °
, All patient	1245.6 (59.5)	49.6 (12.1)	35.6 (3.5)	196.7 (7.8)	5.3 (2.2)	3.7 (1.9)	1.8 (0.3)	99.4 (9.8)	77.8 (11.3)	23.8 (20.5)
Group A	1180.6 (52.8)	43.7 (10.4)	32.6 (4.5)	207.3 (8.1)	5.6 (2.7)	3.6 (1.2)	1.9 (0.8)	97.9 (10.7)	73.7 (10.3)	20.9 (19.8)
Group B	1282.3 (54.5)	52.2 (11.5)	37.6 (5.5)	186.7 (7.8)	5.1 (2.1)	3.8 (2.1)	1.7 (0.7)	109.4 (11.7)	84.6 (7.9)	29.7 (17.8)
*P* value	.0015	<.0001	< 0.001	.093	0.367	0.449	0.425	0.03	0.04	0.813

Moreover, the mean MCSA was 196.7° in all subjects, 207.3° in group A, and 186.7° in group B. The mean CSA was significantly different between the two groups (*p* < 0.001); however, no statistically significant difference in MCSA was observed (*p* = 0.093). The minimum CSA was 35.6 mm^2^ in all subjects, 32.6 mm^2^ in group A, and 37.6 mm^2^ in group B. Minimum CSA was significantly different between the two groups (*p* < 0.001).

The mean of the maximum CTD was 5.3 mm in all subjects, 5.64 mm in group A, and 5.1 mm in group B. The mean of the minimum CTD was 3.7 mm in all subjects, 3.6 mm in group A, and 3.8 mm in group B. The range of CTD was defined as the difference between the maximum and minimum CTD; the mean range of CTD was 1.9 mm in group A and 1.7 mm in group B. No significant differences in maximum CTD (*p* = 0.367), minimum CTD (*p* = 0.449), or range of CTD (*p* = 0.425) between the two groups were found.

The mean of the maximum CTA was 99.4° in all subjects, 97.9° in group A, and 109.4° in group B. The mean of the minimum CTA ​​was 77.8° in all subjects, 73.7° in group A, and 84.6° in group B. Significant differences in maximum CTA (*p* = 0.03) and minimum CTA (*p* = 0.04) were observed. The range of CTA was defined as the difference between the maximum and minimum CTA; the mean range of CTA was 20.9° in group A and 29.7°. No significant difference between the two groups was noted (*p* = 0.813).

## Discussion

This study demonstrated that CT could be a useful tool for cubital tunnel measurement in idiopathic CuTS. The goal of this study was to investigate the bony structure of the cubital tunnel involved in idiopathic CuTS. Several previous investigations suggested that decreased CTV and increased cubital tunnel pressure are causative factors of CuTS [7, 21–24]. Although space-occupying lesions or thickened cubital tunnel retinaculum is known to be responsible for the changes in the volume or pressure of the cubital tunnel, we hypothesized that anatomical variation of the bony structure (size and shape of the cubital tunnel) may also affect the volume or pressure of the cubital tunnel. In this study, we proposed a new bony cubital tunnel with a new boundary. Bony size and shape of the cubital tunnel have been implicated in ulnar nerve compressive neuropathy symptoms (small volume or CSA, and narrowed dimensions indicating increased hydrostatic pressure and mechanical impingement).

CTV, CSA, CTD, and CTA are the most accepted and widely used radiographic parameters for diagnosing both carpal tunnel syndrome and CuTSs. These parameters have been used in previous studies, and most of these parameters were measured using simple radiographs, ultrasonography, or MRI [25, 26]. Nevertheless, CT could be used to measure bony structures more accurately and at multiple levels. Therefore, CSA, CTD, and CTA measurement using CT axial images would be relevant. However, traditional axial images (axial images obtained along the axis of the upper arm in the elbow extension state) do not properly reflect the curvature of the cubital tunnel. Thus, the radiographic parameter could be underestimated or overestimated. To resolve the discrepancies due to tunnel and elbow orientations, axial images reconstructed using an axis of bony cubital tunnel were used. This reconstruction images allowed improved visualization of the cubital tunnel anatomy and better quantification of the parameters compared to traditional axial images.

In this study, the volume of the 3D modeling of the cubital tunnel was measured using Materialize Mimics software and was compared between group A and group B; we found that the CTV could be related to idiopathic CuTS. Moreover, the CTV was significantly smaller in group A (*p* = 0.015), which suggested that a small CTV is related to the development of idiopathic CuTS. These results supported the hypothesis that idiopathic CuTS could be attributed to the formation of relatively raised hydrostatic pressure due to small CTV, even in the absence of space-occupying lesion or thickened cubital tunnel retinaculum. Currently, no studies on the relationship between the structure of the bony cubital tunnel and the occurrence of symptoms have been conducted.

The difference between the MCSA and minimal CSA suggests that the nerve compression due to the osseous structure is most likely to be the most severe. The angle at which the MCSA is at the minimum corresponds to the vertex of the medial epicondyle, which indicates that nerves are prone to impingement at this site.

Furthermore, CTD and CTA provide additional information about the shape of the cubital tunnel. Previous studies showed that the greater the CTD value or the smaller the CTA value, the higher the incidence of idiopathic CuTS [23, 27]. CTD was not significantly different between the two groups in this study; however, a significant difference between maximum CTA (*p* = 0.03) and minimum CTA (*p* = 0.04) was found. This indicates that the portion of the cubital tunnel where the acute angle is formed is likely to influence the occurrence of idiopathic CuTS, which further suggests that mechanical impingement may occur because of the acute angle or that the ulnar nerve at the acute angle may be subjected to continuous tension; the CTA in CuTS in our study is consistent with that in previous studies [28].

The range of CTA indicates the amount of change in the angle of the base of the cubital tunnel. A greater range of CTA indicates that the angle formed by the base changes more sharply. However, unlike maximum and minimum CTA, the range of CTA did not show a significant difference in both groups (*p* = 0.813), suggesting that the degree of CTA change in the cubital tunnel was not associated with the occurrence of symptoms. Different from previous studies, our study did not use cadavers and subjects of different ages were included, which granted an advantage of identifying relationship with clinical symptoms.

We focused on the relationship between idiopathic CuTS and the bony structure of the cubital tunnel. We analyzed the cubital tunnel based on the axis of bony cubital tunnel. The volume, CSA, MCSA of the bony cubital tunnel and maximum and minimum values ​​of the CTD and CTA were evaluated and compared. Based on the results, we could assume that the smaller the CTV, the smaller the CSA, and the more acute the CTA, the more the ulnar nerve gets compressed.

This study has some limitations. First, the number of patients was small. Second, the nerve size is not directly measured since CT could not directly measure the size of the nerves, it would be of great significance to study the relationship between the cross-sectional area of the nerves measured using Ultrasound and CT measured bony structures. Third, only the bony structure was analyzed; thus, the influence of soft tissues on nerve compression was not investigated. Hence, measurements of volume, shape of the section, and configuration (angle and depth) of the actual course of the ulnar nerve in this study differed from those of previous studies. Thereby, it was able to find factors that might affect ulnar nerve compression. As a consequence, the bony cubital tunnel was redefined and analyzed in association with causes generating clinical symptoms.

## Conclusions

Idiopathic CuTS has been elucidated. In this study, we could confirm that the shape of the bony cubital tunnel is an important cause of CuTS, and the normal variation of CTV and CTA and CSA of ​​the cubital tunnel could also influence the occurrence of idiopathic CuTS.

## Data Availability

The datasets used and/or analyzed during the current study are available from the corresponding author on reasonable request.

## Abbreviations

3D: Three dimensional
ABT: Axis of bony cubital tunnel
AME: apex of the medial epicondyle
CSA: Cross-sectional area
CT: Computed tomography
CTA: Cubital tunnel angle
CTB: center of the trochlear border
CTD: Cubital tunnel depth
CTV: Cubital tunnel volume
HU: Hounsfield unit
MCSA: Minimal cross sectional area angle
MRI: Magnetic resonance imaging
